# 片段/虚拟分子印迹聚合物的应用新进展

**DOI:** 10.3724/SP.J.1123.2020.08008

**Published:** 2021-02-08

**Authors:** Yixiao WANG, Jinhua LI, Liyan WANG, Ji QI, Lingxin CHEN

**Affiliations:** 1.中国科学院烟台海岸带研究所, 中国科学院海岸带环境过程与生态修复重点实验室, 山东省海岸带环境过程重点实验室, 海岸带环境工程技术研究与发展中心, 山东 烟台 264003; 1. Chinese Academy of Sciences Key Laboratory of Coastal Environmental Processes and Ecological Remediation, Shandong Key Laboratory of Coastal Environmental Processes, Research Center for Coastal Environmental Engineering and Technology, Yantai Institute of Coastal Zone Research, Chinese Academy of Sciences, Yantai 264003, China; 2.中国科学院大学资源与环境学院, 北京 100049; 2. School of Source and Environment, University of Chinese Academy of Sciences, Beijing 100049, China

**Keywords:** 分子印迹聚合物, 片段印迹, 虚拟模板印迹, 制备, 应用, 综述, molecularly imprinted polymers (MIPs), fragment imprinting, dummy template imprinting, preparation, application, review

## Abstract

分子印迹聚合物(MIPs)是通过模拟酶与底物或抗原抗体特异性结合原理而制备的高分子聚合物,以其结构预定性、识别特异性、制备简便、成本低、耐受性强等优点而被广泛用于样品前处理、传感分析、生物医药、环境/食品分析等多个领域。目前已发展多种策略用于MIPs制备,达到简化制备过程或提高聚合物性能等目的,极大拓宽了MIPs的应用范围。对各种先进印迹策略及其组合使用的探索已成为MIPs制备的研究热点之一。其中,片段印迹策略和虚拟模板印迹策略备受青睐。片段印迹策略是选择目标分子中含有特定官能团的一部分(片段结构)作为模板进行印迹,通过对片段的识别达到对整个分子的识别,能够克服某些目标物不易获得或体积较大不适合作为模板的问题,为印迹易失活、易传染的目标物及整体印迹困难的大分子提供可行的方法。虚拟模板印迹策略是选用与目标物特异性结构相似或相同的其他物质代替目标物作为模板制备MIPs,可在很大程度上解决模板不易获得或较昂贵等问题,以及避免模板可能泄漏对结果造成的影响,尤其适用于目标物造价高、具有感染性、易燃易爆、易降解等不适合作为模板分子的情况。该文选取了最近4年发表在ACS、Elsevier、RSC等数据库约20篇相关文献,综述了片段/虚拟MIPs(FMIPs/DMIPs)的应用新进展。首先,针对蛋白质和微生物检测以及哺乳动物细胞印迹,介绍了FMIPs在生物医药领域的应用,另外介绍了FMIPs在食品分析领域的研究进展。随后,介绍了DMIPs在样品前处理和传感分析领域的应用。在样品前处理中,DMIPs主要作为固相萃取吸附剂进行装柱固相萃取、分散固相萃取、磁固相萃取、基质固相分散萃取等,或作为分子印迹膜材料,用于选择性萃取和富集分离样品中的目标分析物。在传感分析领域,DMIPs主要作为传感器的传感和转导元件,提高化学发光或荧光检测等方法的灵敏度和准确度。最后,对片段印迹和虚拟模板印迹策略的优缺点、区别与联系进行了总结,并展望了这两种策略的发展与应用前景。

## Abstract (en)

Molecularly imprinted polymers (MIPs) are designed to mimic the specific binding principle of enzymes to substrates or antigens to antibodies, while holding several advantages such as structure predictability, recognition specificity, easy preparation, low cost, high physical robustness, and thermal stability. Therefore, they have been widely applied in many fields including sample preparation (pretreatment), sensing analysis (chemo/biosensors), biomedicine, and environment/food analysis. To date, several strategies were developed for MIPs preparation, aiming to simplify the preparation process and/or improve the properties of the polymers, greatly broadening its usability. The exploration in various advanced imprinting strategies and their combinational use has become a research hotspot in MIPs preparation, among which the fragment imprinting strategy and the dummy template imprinting strategy are especially favored. Fragment imprinting, also called segment imprinting, uses a partial structure of the target molecule as a pseudo-template to prepare MIPs. This strategy is useful to target molecules that are not easy to obtain or that are too large to be used as templates, providing a feasible method for imprinting target analytes that are easy to inactivate or infect, as well as macromolecules that are difficult to imprint. In turn, dummy template imprinting uses molecules with structure, shape, and size similar to the target analytes as templates for imprinting. Because the target is not directly used as a template, this strategy can overcome problems of template leakage, as well as solve target molecule-related difficulties as they can be expensive, infectious, flammable, explosive, or chemically instable. This mini-review compiles information of several articles published in the last four years across ACS, Elsevier, RSC, and other databases, summarizing the most recent advances in the application of fragment/dummy template MIPs (FMIPs/DMIPs). Herein, the biomedical application of FMIPs is mainly addressed as a strategy for the detection of proteins and microorganisms, and the application of FMIPs in the field of food analysis is also explored. In recent years, the imprinting of mammalian cells has made some progress in the application of FMIPs. Mammalian cells, especially cancer cells, overexpress some proteins and sugars, which are good fragment templates. Consequently, the fragment imprinting strategy is widely used in cancer cell imaging, localization, and treatment. Moreover, due to the complicated structure and easy inactivation of some proteins, their MIPs are often prepared by fragment imprinting (also called epitope imprinting). As some microorganisms are infectious, imprinting microorganisms directly can pose a risk; therefore it is safer to also use the fragment imprinting strategy in such cases. The recent application of fragment imprinting strategy in other areas remains scarce. Nonetheless, three studies in the food analysis have explored this possibility. DMIPs are widely used in sample pretreatment and sensing analysis, and they are mainly used as SPE adsorbents for packed SPE, dispersive SPE (DSPE), magnetic SPE (MSPE), and matrix solid phase dispersion (MSPD) extraction. In addition, DMIPs are employed as molecularly imprinted membrane materials. As a result, by virtue of DMIPs, selective extraction and enrichment of target analytes from complicated samples can be achieved. MIP-based sensors can either recognize or transduce, meaning that they can specifically recognize and bind target analytes as well as generate output signals for detection. Because of the high selectivity of MIPs, the use of a dummy template imprinting strategy solves the problem of template leakage in the process of recognition and adsorption, further improving the detection accuracy and sensitivity of the sensor. These features expand the application range of MIP-based sensors. This review briefly overviews the construction and application of chemiluminescence and fluorescence sensors based on DMIPs. Lastly, the advantages and disadvantages, differences, and relationships among the two strategies are summarized. Despite of their potential, four main challenges still remain as major setbacks for the application of FMIPs and DMIPs: (i) the difficulty to select or prepare appropriate fragment templates and dummy templates; (ii) how to ensure that there is almost no difference in the recognition adsorption selectivity between the fragment/dummy template and the original template, so as to ensure optimal recognition specificity; (iii) the use of, environment-friendly reagents to reduce pollution during FMIPs/DMIPs preparation and use to conform with green chemistry requirements; (iv) how to strengthen the industrial and commercial applications of FMIPs and DMIPs. Therefore, significant efforts should be made to develop new imprinting strategies and techniques, as well as to adopt combinational imprinting approaches for FMIPs/DMIPs preparation to expedite the sustainable development and efficient application of FMIPs and DMIPs.

分子印迹聚合物(MIPs)是通过模拟酶与底物或抗原抗体特异性结合原理制备的高分子聚合物,因其识别特异性、结构预定性、易制备、耐受性强等优点而被广泛应用^[1]^。目前已发展多种印迹策略用于MIPs制备,提高了选择吸附性能,扩展了应用。其中,片段分子印迹聚合物(FMIPs)策略是通过选取目标物的一段特异性识别序列作为模板用于合成MIPs,克服了某些目标物不易获得或体积较大不适合作为模板的问题,尤其对于易失活的生物大分子及微生物的印迹意义重大;虚拟模板分子印迹聚合物(DMIPs)策略是选用与目标物的特异性结构相似或相同的其他物质,替代目标物作为模板制备MIPs,可在很大程度上解决模板不易获得或较昂贵等问题,以及避免识别吸附过程中模板可能泄漏,降低对分析造成的影响。本文主要综述了片段模板MIPs在生物医药领域、虚拟模板MIPs在样品前处理和传感分析领域的最新研究动态和典型应用。

## 1 片段印迹策略

片段印迹策略是在MIPs制备过程中,用目标化合物的一部分代替整个目标化合物作为模板。某些目标化合物尤其是生物大分子或细菌、病毒等微生物,具有不易获得、性质不稳定或毒性强等特点,因此无法用完整的靶分子作为模板制备MIPs。使用靶分子中的一段特异性片段代替靶分子作为模板制备出FMIPs的片段印迹策略可很大程度上解决上述问题,并可简化制备步骤,提高聚合物性能。片段印迹多应用于生物医药领域,尤其是蛋白质和微生物检测方面,对哺乳动物细胞印迹的研究也有进展,此外,FMIPs在食品分析领域也有应用。

### 1.1 生物医药

片段印迹策略为整体印迹有困难、易失活或不易保存的目标物的印迹提供了可行的方法。近年来,片段印迹策略的研究工作集中在与大分子密切相关的生物医药领域,主要包括蛋白质印迹、微生物印迹和哺乳动物细胞印迹。

1.1.1 蛋白质印迹

蛋白质相对分子质量大,结构复杂,对外界条件变化敏感,较易失活,不易溶于有机溶剂,不易与MIPs识别结合,这些都增加了制备蛋白质MIPs的难度。片段印迹策略的使用,很大程度降低了蛋白质MIPs制备过程的难度,提高了MIPs对大分子印迹的能力。在蛋白质MIPs制备过程中,片段印迹策略通常是指将蛋白质的一段特异性氨基酸序列作为整个蛋白质识别的模板,这些特异性部分又称作抗原决定簇或表位,因此也称作表位印迹策略^[2]^。

研究人员^[2,3]^开展了系列表位印迹策略制备蛋白质MIPs的研究。例如,以P32膜蛋白(4T1癌细胞表面过表达的膜蛋白)N端表位为主要模板、阿霉素(DOX)为次要模板,采用沉淀聚合反应制备了FMIPs纳米颗粒^[2]^。该FMIPs的DOX印迹腔可稳定携带药物,保持药物活性,表位印迹腔可使FMIPs特异性识别P32膜蛋白。如图1所示,该实验选取一段*α*-螺旋蛋白作为表位模板,制备的荧光FMIPs可以搭载药物并可特异性识别表面具有*α*-螺旋蛋白的癌细胞,即4T1癌细胞,可通过荧光成像对癌细胞进行定位。对荷瘤小鼠的治疗中,该FMIPs@DOX的治疗效果远高于普通药物的治疗效果,具有良好的临床应用价值。随后,他们又以人血清白蛋白(HSA)的C端肽和转铁蛋白(Trf)的C端肽为表位模板制备了一种热敏感的双模板表位FMIPs,能够同时识别HSA和Trf^[3]^。采用金属螯合印迹和蒸馏沉淀的方法,以丙烯酸锌和异丙基丙烯酰胺为功能单体,制备了FMIPs。该方法制备时间短,仅需30 min。FMIPs对样品中的HSA和Trf均有良好的识别吸附能力,最大吸附量分别为103.67 mg/g和68.48 mg/g,印迹因子分别为2.57和2.17,且具有热敏性能,能够实现温控吸附和释放靶蛋白。

**图1:**
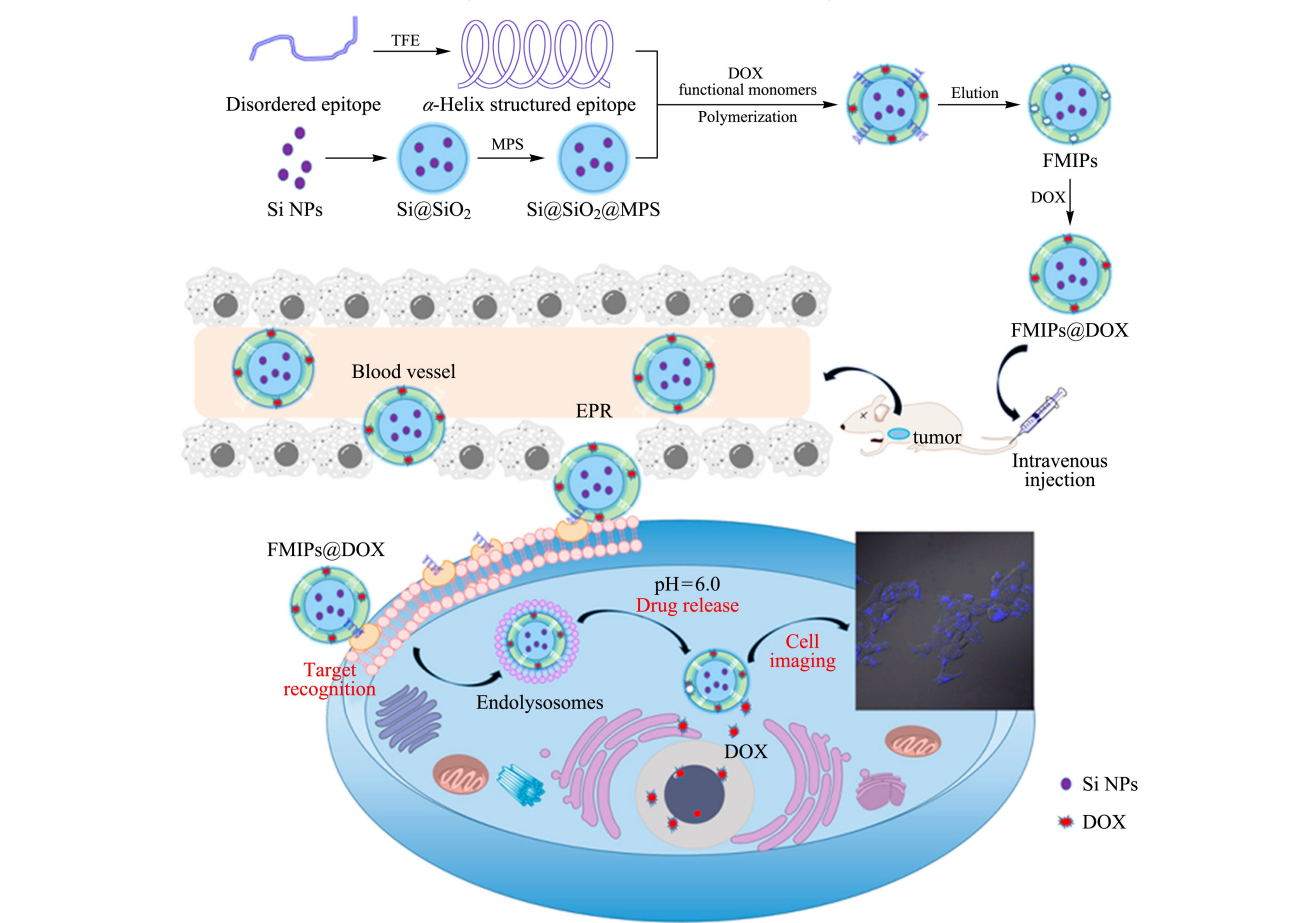
FMIPs的合成路线及装载DOX的FMIPs在肿瘤治疗上的应用^[2]^

1.1.2 微生物印迹

以细菌为模板制备MIPs通常是将整个细菌作为模板制备全细胞受体检测聚合物;然而,大部分细菌虽有细胞壁的支撑但仍具有形变特点,无法满足印迹腔单一的刚性结构要求,在制备及后续识别吸附上有困难,且如果操作不当容易被感染。片段印迹策略利用细菌的特异性片段(如糖蛋白、聚糖等)作为模板进行印迹,可创造更多的结合位点,降低制备难度,优化制备工艺,并且安全可靠。

对微生物片段印迹策略的应用多是针对其特有的蛋白质表位进行的。Gupta等^[4]^构建了一种检测脑膜炎患者血样中奈瑟菌的电化学石英晶体微天平(EQCM)传感器。以存在于脑膜炎奈瑟菌中的铁结合蛋白(ftp A)为模板,在EQCM的镀金表面通过自组装反应制备了单层膜,利用硫醇键将模板蛋白锚定到单层膜上,形成分子印迹膜。该分子印迹膜用于脑膜炎患者实际血样检测时,对ftp A蛋白的检出限为1.39 ng/mL,印迹因子为12.27,表明对模板分子具有优异的识别和结合能力。相比于全细胞受体聚合物的识别检测,该方法为快速检测脑膜炎提供了一种可靠、简便、成本低的诊断工具,临床应用潜力巨大。Kushwaha等^[5]^发展了一种EQCM传感器用于检测麻风分歧杆菌。利用麻风分歧杆菌表面蛋白的表位序列作为模板,3-甲基丙烯酸磺丙酯钾盐、甲基丙烯酸苄酯和4-氨基苯硫酚作为功能单体,合成的FMIPs能够与感染患者血样中带有表位序列的麻风分歧杆菌特异性结合,而不结合其他血浆蛋白。该FMIPs传感器对实际样品中麻风分歧杆菌的检出限和定量限分别低至0.161 nmol/L和0.536 nmol/L,为早期预防及快速诊断麻风病提供了有效手段。

病毒感染性强,对生命健康危害较大。以病毒为模板的MIPs快速检测方法,在疾病预防和诊疗方面有很大的应用潜力。但对于病毒MIPs材料的制备存在很多问题,比如病毒在外界环境下较脆弱、易失活,实验人员操作不当有感染风险等。通过片段印迹策略,选取病毒表面能够代表整个病毒的一段特异性序列作为模板,可以很大程度上解决上述问题。Chou等^[6]^从人类免疫缺陷病毒(HIV)蛋白酶中选取螺旋表位肽作为模板,并将肽模板与水、三氟乙醇和乙腈按一定比例混合,以扩大溶液中的螺旋构象,在石英晶体微天平芯片上制备螺旋表位介导的MIPs。该MIPs对HIV蛋白酶有很高的亲和力,所构建的传感器对HIV蛋白酶的检出限为0.1 ng/mL,提供了一种临床检测病毒的可行方法,这项技术有望结合HIV抑制剂对HIV病毒进行靶向治疗,前景广阔。

1.1.3 哺乳动物细胞印迹

相比于细菌细胞的细胞壁支撑作用,哺乳动物细胞的细胞膜较脆弱,因此传统的整体印迹方法,直接印迹动物细胞需要更温和的条件,甚至需要对细胞进行一定的前处理。相较于对哺乳动物细胞的整体印迹技术,片段印迹策略是利用哺乳动物细胞膜上有丰富的受体,包括糖蛋白、糖脂等物质。不同的细胞类型表达不同的蛋白质、糖类等,以这些物质为模板制备FMIPs,需要注意的是用于细胞的MIPs要具有较好的生物相容性,另外,片段印迹策略制备的FMIPs大小应与体内大分子相近,以保证其在血管、淋巴系统等组织中的扩散和循环。

癌细胞表面会表达大量的特异性糖蛋白、糖脂及其他化合物,因此片段印迹策略在癌细胞的靶向定位及成像上有着丰富的应用。除上文提到的以蛋白质表位作为模板制备的FMIPs可以作为递药系统对癌细胞定位、治疗、成像外,多糖以及酸类物质也是重要的片段印迹策略的模板。Liu等^[7]^以唾液酸为模板,设计合成了一种苯基硼酸基团修饰改性的荧光FMIPs。使用该聚合物分别对表面过度表达唾液酸的DU145癌细胞,以及表面未过度表达唾液酸的HeLa细胞进行检测。实验结果表明,FMIPs对DU145癌细胞表现出选择性染色,且荧光强度良好,但即使在长时间的孵育后,FMIPs也没有进入HeLa细胞。这种方法特异性高,其荧光特性可以准确地对癌细胞进行定位。普通的血型鉴别通常是基于抗体与血细胞表面的抗原识别结合产生凝集反应的原理,通过FMIPs特异性识别结合血细胞表面的抗原区分不同血型的方法,更加简便快捷,且避免了抗体作为一种蛋白质活性易受外界环境因素影响的缺点。抗原决定簇A和B是存在于人类红细胞膜上的两种最重要的抗原,且他们均由寡糖分子决定类型。Piletsky等^[8]^以半乳糖、岩藻糖和半乳糖组成的B型三糖作为模板制备了磁性分子印迹纳米颗粒。具有顺磁性的FMIPs会将已特异性识别结合的血细胞吸引到磁铁上,导致样本褪色,从而进行鉴别。这项研究证明了FMIPs在鉴别血型上的可行性,但同时鉴别4种血型还存在一定困难。

### 1.2 食品分析

片段印迹策略除在生物医药领域有大量应用外,在食品分析领域也有应用,但在其他领域的最新应用鲜有报道。如以D-葡萄糖为片段模板选择性提取大豆中的三种黄酮苷^[9]^,以没食子酸为片段模板从茶叶中萃取酯基儿茶素^[10]^,以邻苯二酚为片段模板萃取茶叶和果汁样品中的多酚类物质^[11]^。Hou等^[11]^以多酚类物质儿茶素、绿原酸和咖啡酸中的共有结构邻苯二酚为片段模板,2-苯胺为自聚合印迹涂层,利用硼酸亲和印迹技术制备了中空多孔结构的FMIPs。中空结构有利于模板分子的去除,并能有效与靶分子结合,2-苯胺自聚合形成的涂层含有丰富的羟基,利于后续材料表面修饰及亲水性提高。该FMIPs作为固相萃取填料高效地选择性识别吸附了茶叶和果汁样品中的上述3种多酚类物质。

## 2 虚拟模板印迹策略

虚拟模板印迹策略是采用在结构、形状和大小及功能上与目标化合物相似的化合物作为模板进行印迹,制备出的聚合物通常称为DMIPs。当原有的模板不易获得,且造价昂贵或有毒时,除用上述的片段印迹策略外,也常用虚拟模板印迹策略,其可以有效避免模板泄漏造成的污染或对目标化合物分析结果造成影响等问题^[12]^。相比于片段印迹策略多用于生物分析,虚拟模板印迹策略适于各类目标物,在样品前处理和传感分析领域应用广泛。

### 2.1 样品前处理

MIPs在样品前处理中,主要作为SPE的吸附剂,即分子印迹固相萃取(MI-SPE),用于选择性萃取、富集、分离样品中的目标分析物。MI-SPE主要包括常规SPE(装柱SPE)、分散固相萃取(DSPE)、磁固相萃取(MSPE)和基质固相分散萃取(MSPD)。Zhang等^[13]^以棉糖代替氨基糖苷类抗生素作为模板分子,甲基丙烯酸为功能单体,三甲基丙烷三丙烯酸酯为交联剂,采用沉淀聚合法制备了新型氨基糖苷类抗生素DMIPs。将此材料作为SPE填料并结合亲水相互作用-高效液相色谱-串联质谱(HILIC-HPLC-MS/MS)技术,成功测定了环境水样中6种痕量氨基糖苷类抗生素(硫酸链霉素、硫酸卡那霉素、硫酸安普霉素、硫酸庆大霉素、妥布霉素和硫酸帕洛莫霉素),富有应用潜力。Chen等^[14]^选择与吡虫啉和啶虫脒具有一段相同结构的烟酰胺作为模板,以聚乙二醇二甲基丙烯酸酯为交联剂、偶氮二异丁腈为引发剂,采用表面印迹技术制备了DMIPs,用作SPE填料,高效富集了茶多酚中的痕量吡虫啉和啶虫脒。他们也分别合成了吡虫啉和啶虫脒作为模板的MIPs,发现DMIPs对这两种农药分子具有良好的吸附能力和选择性。相比于传统填料,MIPs填料以其高选择性极大增强了SPE的富集效率,进而提高了后续分析的准确度。

近来,DMIPs在MSPE^[15,16]^和MSPD^[17]^中的应用持续增加。Zhang等^[17]^采用Pickering乳液聚合法以*α*-(2,4-二氯苯基)-1*H*-咪唑-1-乙醇(DCE)为模板合成了DMIPs,作为MSPD吸附剂用于鱼样品中唑类杀菌剂(攀缘唑、克霉唑和咪康唑)的前处理。MSPD萃取时间短,通过将DMIPs与样品充分研磨,萃取剂与目标物可充分接触,从而大大提高吸附萃取效果。

分子印迹膜(MIM)技术是一种将MIPs合成在多孔膜表面的技术。MIM具有合成简便、成本低、绿色环保等优点,可以作为一种样品前处理材料,从复杂基质中分离富集目标物。通过虚拟模板策略,可以克服MIM在使用过程中产生的模板泄漏问题,避免对后续检测带来影响,极大提高了MIM的性能和应用。田红静等^[18]^在聚偏氟乙烯膜表面,以环丙沙星的结构类似物恩诺沙星为虚拟模板、甲基丙烯酸为功能单体、乙二醇二甲基丙烯酸酯为交联剂制备了恩诺沙星MIM,萃取牛奶样品中的环丙沙星,结合仪器分析,快速检测了牛奶中痕量环丙沙星残留。将虚拟模板印迹策略与MIM相结合的制备方法为快速准确检测复杂样品中痕量目标物残留提供了新思路。

### 2.2 传感分析

传感器主要由信号识别单元和信号转换单元组成,基于MIPs的传感分析,是指MIPs在传感器中作为信号识别单元,特异性识别结合分析物后产生一定的信号,产生的信号被识别后得到分析数据。虚拟模板印迹策略的使用,一定程度上解决了MIPs在识别吸附目标物的过程中模板泄漏的问题,进一步提高了传感器的检测准确度和灵敏度,拓展了传感器的应用范围。

Li等^[19]^以磺胺苯(SZ)为虚拟模板,采用本体聚合法制备了能够同时识别15种磺胺类药物的DMIPs,将其作为识别试剂在常规的96孔微板上构建化学发光传感器。传感器可用于测定猪肉中15种磺胺类药物的残留量,检出限为1.0~12 pg/mL,响应时间在30 min内,可重复使用4次以上。该传感器可以大量筛查样本,且操作简单,选择性强,灵敏度高,在快检领域应用潜力巨大。

虚拟模板印迹策略也常与其他印迹策略相结合进行传感分析。Huang等^[20]^将双模板印迹策略和虚拟模板印迹策略结合,制备了MIPs化学发光传感器,用于鸡肉样品中除虫菊酯类农药的检测。以除虫菊酯I和苯基醚为双模板合成了用于除虫菊酯类识别的双-虚拟模板MIPs (DDMIPs)。将DDMIPs颗粒涂覆在96微板孔中,然后加入咪唑增强的双(2,4,6-三氯苯酚)草酸-H_2_O_2_系统来诱导光信号。最后,利用化学发光强度的变化用于分析物定量测定。结果表明,该传感器可重复使用4次。10种除虫酯类农药的检出限为0.3~6.0 pg/mL,空白鸡肉样品的加标回收率为70.5%~99.7%。Guo等^[21]^利用量子点量子产率高、光稳定性好等优点制备了量子点分子印迹荧光传感器。以5,7-二甲氧基香豆素为虚拟模板,通过Stöber法制备了包覆CdTe量子点的DMIPs(MIP@CdTe QDs),利用量子点的发光特性制备了荧光传感器,用于对黄曲霉毒素B1(AFB1)的快速特异性识别。研究表明,该传感器与传统的UPLC-MS测定的结果无显著差异。该DMIPs传感分析方法有望为快速检测复杂基质中痕量外源有害物质开辟新途径。

## 3 片段印迹和虚拟模板印迹策略的比较

片段印迹和虚拟模板印迹策略都不直接采用待测目标物作为模板,在一定程度上解决了MIPs使用过程中识别吸附不稳定、模板泄漏、某些模板不易获得等问题,极大拓展了MIPs的应用范围。本文对两种策略的异同点及优缺点进行了总结(见图2)。片段印迹策略对以细菌和病毒(传染性强的物质)为模板的印迹过程操作条件更安全,因使用的模板尺寸较小,有利于创造更多印迹位点。虚拟印迹尽管解决了痕量分析中的模板泄漏问题,但有可能会导致识别选择性降低,进而降低萃取效率。当选取的模板分子与目标物的某部分特定结构相同,但模板分子又是另一种不同的物质时,既可认作片段印迹策略也可认作虚拟模板印迹策略。若根据一类目标物的共有结构,筛选出理想的模板分子用于制备FMIPs/DMIPs,即可对一类具有共同结构的物质进行同时、选择性富集和分离测定,实现高通量分析。

**图2:**
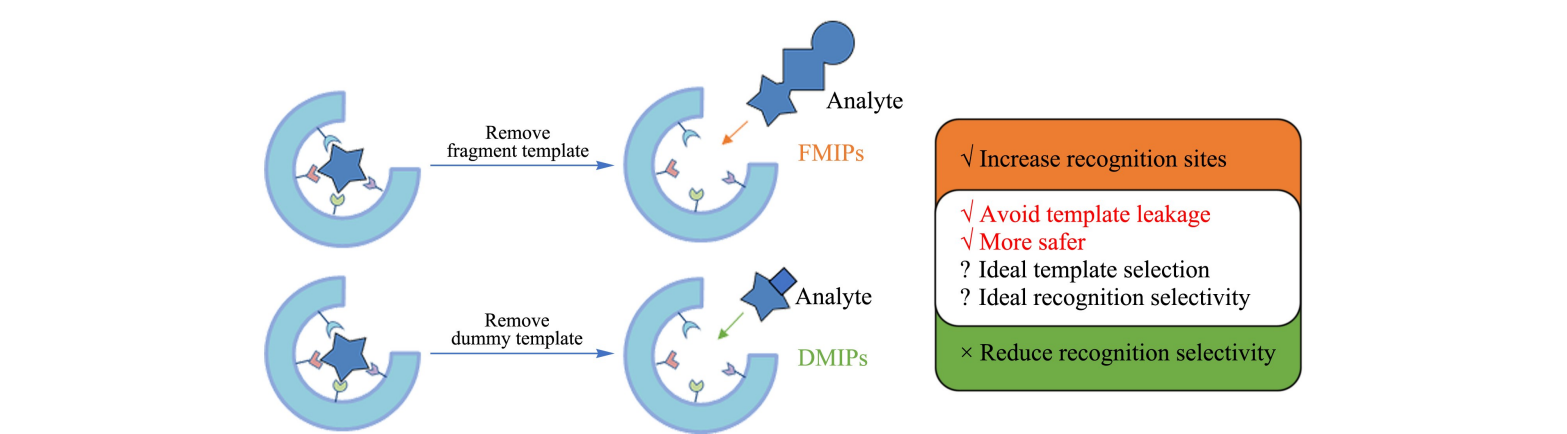
片段印迹策略和虚拟模板印迹策略的基本示意图及比较

## 4 结论与展望

尽管片段印迹策略和虚拟模板印迹策略在MIPs制备过程中应用广泛,然而,目前仍存在以下4点主要问题亟待解决:在策略使用过程中,选择或制备合适的片段模板和虚拟模板仍然有困难^[22]^;如何确保制得的模板与原模板在吸附选择性上几乎没有差异,确保理想的特异识别性;如何在制备和应用过程中使用环保试剂,减少环境污染,实现绿色化学的目标^[23]^;通过实验得到的材料如何进一步产业化,提高实用效果,以促进生产力发展。期待通过发展更多的制备策略和技术及其组合使用,能够更快更好推进FMIPs/DMIPs在更多领域持续发展和高效应用。
